# Sensitisation of an Azole-Resistant *Aspergillus fumigatus* Strain containing the Cyp51A-Related Mutation by Deleting the SrbA Gene

**DOI:** 10.1038/srep38833

**Published:** 2016-12-09

**Authors:** Daisuke Hagiwara, Akira Watanabe, Katsuhiko Kamei

**Affiliations:** 1 Medical Mycology Research Center, Chiba University, 1-8-1, Inohana, Chuo-ku, Chiba City, 260-8673, Chiba, Japan

## Abstract

Azoles are widely used for controlling fungal growth in both agricultural and medical settings. The target protein of azoles is CYP51, a lanosterol 14-α-demethylase involved in the biosynthesis of ergosterol. Recently, a novel azole resistance mechanism has arisen in pathogenic fungal species *Aspergillus fumigatus*. Resistant strains contain a 34-bp or 46-bp tandem repeat (TR) in the promoter of *cyp51A*, and have disseminated globally in a short period of time. In this study, we investigated whether an azole-resistant strain with a 46-bp TR (TR46/Y121F/T289A) could be sensitised to azoles by deletion of *srbA*, encoding a direct regulator of *cyp51A*. The loss of SrbA did not affect colony growth or conidia production, but decreased expression of *cyp51A*. The *srbA* deletion strain showed hyper-susceptibility to medical azoles as well as azole fungicides, while its sensitivity to non-azole fungicides was unchanged. This is the first demonstration that deletion of a regulator of *cyp51A* can sensitise an azole-resistant *A. fumigatus* strain. This finding may assist in the development of new drugs to help combat life-threatening azole-resistant fungal pathogens.

Filamentous fungi (moulds) are ubiquitous in nature, and as well as causing significant crop damage, they can be pathogenic for humans. To prevent harmful fungal growth, a variety of antifungal chemicals are used to protect wood, plant materials, crops, food, and human health. Azoles are antifungals that are widely used in both agricultural and public health settings because of their excellent activity against a wide spectrum of plant and human pathogenic fungi. However, increasing levels of azole resistance have been noted over the last decade^1^,^2^. The situation is further complicated by the finding that some isolates of the major human pathogenic fungus *Aspergillus fumigatus* have acquired cross-resistance to medical azoles through exposure to azole fungicides in the environment^3^,^4^.

The target molecule of azoles is CYP51, a lanosterol 14-α-demethylase involved in the biosynthesis of ergosterol, which is an essential fungal membrane lipid. *A. fumigatus* has two Cyp51 proteins, Cyp51A and Cyp51B, inhibition of which by azoles results in a significant change in sterol profile in the cells. Ergosterol deficiency as well as production of toxic intermediates is thought to cause the antifungal effect^5^. Expression of *cyp51A* and *cyp51B* is regulated by a sterol element binding protein (SREBP), SrbA, in *A. fumigatus*^6^,^7^. A *srbA* deletion mutant showed decreased levels of *cyp51A* and *cyp51B* expression, as well as hyper-sensitivity to medical azoles such as itraconazole, voriconazole and posaconazole. It has been clearly demonstrated that SrbA regulates *cyp51A* expression in a direct manner; thus, SrbA is the only known direct regulator of intrinsic azole resistance among fungi^8^. Notably, the *srbA* deletion mutant showed impaired growth under hypoxic conditions, suggesting that proper regulation of the ergosterol biosynthesis pathway is necessary for fungi to adapt to oxygen limitation.

Azole-resistant *A. fumigatus* strains, with a combination of specific amino acid substitutions and a 34-bp or 46-bp tandem repeat (TR) in the promoter region of *cyp51A* (TR34/L98H and TR46/Y121F/T289A), have been isolated from patients regardless of azole treatment history, as well as from the environment^3^,^9^,^10^,^11^,^12^,^13^,^14^. It is widely accepted that these resistant strains are derived from exposure to azole fungicides in the environment^4^. This acquired azole resistance mechanism limits the choices for effective drug therapy of aspergillosis caused by *Aspergillus* fungi. Alarmingly, strains with the TR34/L98H and TR46/Y121F/T289A genotypes have rapidly spread across the globe since first reported in 2007 and 2012, respectively. The most recent epidemiological report from the Netherlands showed that 225 (23.6%) and 98 (10.3%) of 952 clinical *A. fumigatus* isolates harboured the TR34/L98H and TR46/Y121F/T289A mutations, respectively^15^. Thus, it is imperative to obtain a better understanding of the mechanisms involved in resistance to develop a strategy for combatting azole-resistant *A. fumigatus* strains.

Genetically reconstituted strains have been used to study the molecular mechanism of the environment-associated resistance mutations^16^,^17^. Strains carrying TR34 or TR46 showed a moderate upregulation (2–2.5-fold) of *cyp51A* expression. This increased expression was partly responsible for the lowered susceptibility to azoles. Notably, similar tandem repeats and insertions in the promoter region of *cyp51* have been reported in several plant pathogens, including *Penicillium digitatum*^18^, *Venturia inaequalis*^19^, *Mycosphaerella graminicola*^20^ and *Monilinia fructicola*^21^. Some of these mutations have been associated with overexpression of *cyp51*. Collectively, these studies have shown that constitutive upregulation of *cyp51A* expression in strains with the tandem repeat or insertion seems to be a prerequisite for the azole resistance. Therefore, in the current study, we examined whether deletion of the direct regulator, SrbA, could reduce *cyp51A* expression in a TR46/Y121F/T289A *A. fumigatus* strain, and whether the deletion would affect susceptibility to azoles. Sensitisation of *A. fumigatus* strains harbouring the widespread resistance mechanisms would have significant implications for the effective control of human and plant fungal pathogens.

## Results

### Construction of the *srbA* deletion mutant

To investigate the role of SrbA in the azole resistant strains, we deleted *srbA* in an *A. fumigatus* strain containing TR46/Y121F/T289A mutations (IFM 63432)^13^. The deletion mutant strain was designated IFM 63432-Δ*srbA*. We obtained two independent isolates (IFM 63432-Δ*srbA* No. 1 and No. 2) from the transformation, and the two isolates showed virtually identical phenotypes. Thus, representative results are shown in the figures. A *srbA* deletion mutant was also successfully constructed in azole-susceptible *A. fumigatus* strain Af293 (designated Af293-Δ*srbA*), and was used as a control throughout the study.

### Colony growth and conidia production in the *srbA* deletion strains

**Figure 1:**
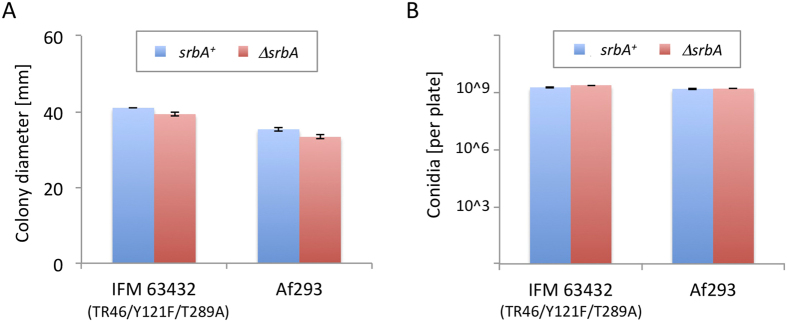
Fungal growth and conidia production. (**A**) Colony diameter was compared between wild-type strains IFM 63432 (TR46/Y121F/T289A) and Af293 and their corresponding *srbA* deletion mutants. All strains were grown on PDA for 48 h. (**B**) The number of produced conidia was compared between strains. Conidia were harvested from strains cultured for 3 days on PDA in 3.5-cm diameter plates. The mean values and standard deviations were obtained from three replicates.

The deletion mutants were assayed for their ability to grow on potato dextrose agar (PDA) (Fig. 1A) and glucose minimal medium (GMM) (data not shown). There were no differences in growth rate between the parental strains (IFM 63432 and Af293) and the *srbA* deletion mutants. The amounts of conidia produced were also comparable between the strains (Fig. 1B).

### qRT-PCR analysis of *cyp51A* expression

**Figure 2:**
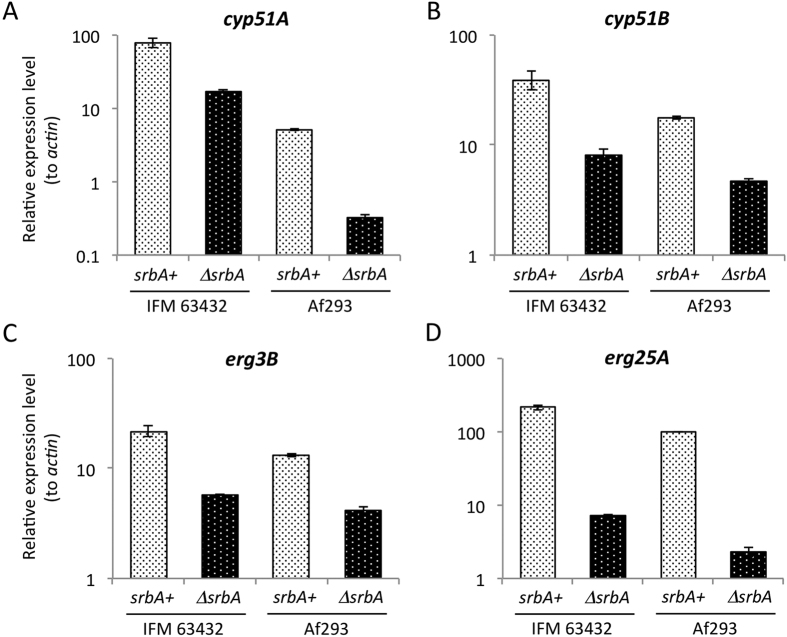
Expression analysis of IFM 63432 and Af293 and the *srbA* deletion mutants. Expression levels of *cyp51A* (**A**), *cyp51B* (**B**), *erg3B* (**C**) and *erg25A* (**D**) were determined by qRT-PCR. The strains were cultivated in PDB for 16 h. The expression levels of the genes of interest were normalised against those of the actin gene. Relative expression ratios are shown. Error bars represent the standard deviations based on three independent replicates.

To assess the role of SrbA in the expression of genes related to azole resistance and/or ergosterol biosynthesis, we investigated the expression levels of *cyp51A* and *cyp51B* by qRT-PCR. As reported previously in other strains^7^,^22^, the levels of *cyp51A* and *cyp51B* expression in the Af293 background were decreased by 93.7% and 73.6%, respectively, in the Δ*srbA* mutant compared with the wild-type (Fig. 2A and B). In the IFM 63432 background, deletion of *srbA* resulted in a reduction of *cyp51A* and *cyp51B* expression of 78.3% and 79.3%, respectively (Fig. 2A and B). It should be noted that *cyp51A* expression was markedly higher (15.3-fold) in IFM 63432 than in Af293, whereas *cyp51B* expression was only 2.2-fold higher in IFM 63432. This suggested that the presence of the TR46/Y121F/T289A mutations might cause increased *cyp51A* expression under the tested conditions.

In addition to Cyp51 genes, other ergosterol synthesis genes (*erg3B* and *erg25A*) are directly regulated by SrbA^7^. Hence, we also examined the expression levels of these genes in the current study. Expression levels of *erg3B* and *erg25A* were 1.7-fold and 2.2-fold higher, respectively, in IFM 63432 than in Af293 (Fig. 2C and D). In IFM 63432, *erg3B* expression was reduced by 73.7% in the Δ*srbA* mutant compared with the parental strain, whereas *erg25A* expression was decreased by 96.7% in the mutant strain. Likewise, in Af293, the Δ*srbA* mutant showed decreases of 68.5% and 97.7% in the expression levels of *erg3B* and *erg25A*, respectively, compared with the wild-type. These results indicated that deletion of *srbA* resulted in a marked reduction in the expression of *cyp51A*, *erg3B* and *erg25A* even in the IFM 63432 background.

### Susceptibility of the IFM 63432-Δ*srbA* strain to azoles

As demonstrated in our previous study, strain IFM 63432 shows multi-azole resistance (minimum inhibitory concentrations (MICs), fluconazole: >64; itraconazole: 2; voriconazole: >8; miconazole: 2; posaconazole: 4 mg/l). In the current study, the susceptibility of IFM 63432-Δ*srbA*, Af293 and Af293-Δ*srbA* to various antifungals was examined using the broth microdilution method (Table 1). While IFM 63432 showed a high MIC for voriconazole, the MIC was markedly lower in the IFM 63432-Δ*srbA* strain. In addition, IFM 63432-Δ*srbA* showed lowered MICs for fluconazole, itraconazole, miconazole and posaconazole compared with the wild-type strain. In the Af293 background, deletion of *srbA* resulted in relatively low MICs for all azoles except fluconazole. The MIC data indicated that SrbA plays a crucial role in azole resistance in strains with the TR46/Y121F/T289A mutations, as well as in Af293. The MICs for amphotericin B and 5-FC, along with the minimum effective concentrations for micafungin and caspofungin, were largely unchanged in the *srbA* deletion strains. The MICs for itraconazole and miconazole were comparable between IFM 63432 and Af293.

**Figure 3:**
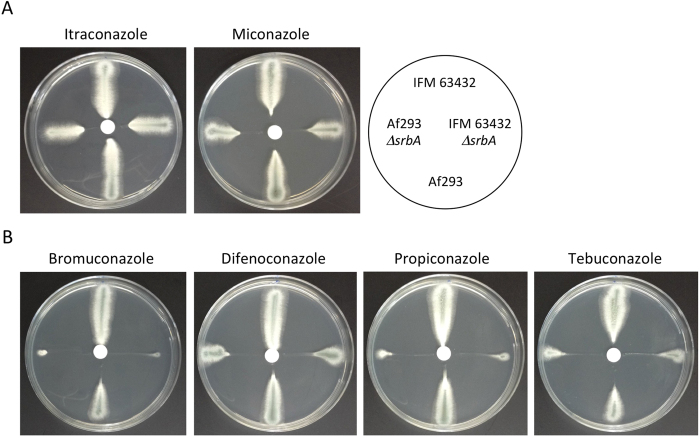
Paper disc diffusion assays. Conidia were streaked on PDA, and a paper disc was placed at the centre of each plate. A 10-μl aliquot of medical azoles itraconazole and miconazole (10 mg/ml) (**A**), or azole fungicides bromuconazole, difenoconazole, propiconazole and tebuconazole (10 mg/ml) (**B**), was spotted onto the paper disc. The plates were then incubated at 37 °C for 24 h before being photographed.

We then examined the levels of growth inhibition by disc diffusion assay. On PDA (Fig. 3A), the parental strains showed similar levels of sensitivity to itraconazole and miconazole. In contrast, IFM 63432 showed much higher levels of resistance to azole fungicides that are widely used in agriculture compared with Af293 (Fig. 3B). As expected, IFM 63432-Δ*srbA* and Af293-Δ*srbA* exhibited hyper-susceptibility to the azole fungicides. Comparing the diameters of the inhibition halos, Af293-Δ*srbA* was more sensitive to the azole fungicides than IFM 63432-Δ*srbA* (Table 2). *srbA* deletion did not affect sensitivity to non-azole fungicides, including iprodione, fludioxonil and kresoxim-methyl, in either of the strain backgrounds (Figure S1).

### Growth under hypoxic conditions

**Figure 4:**
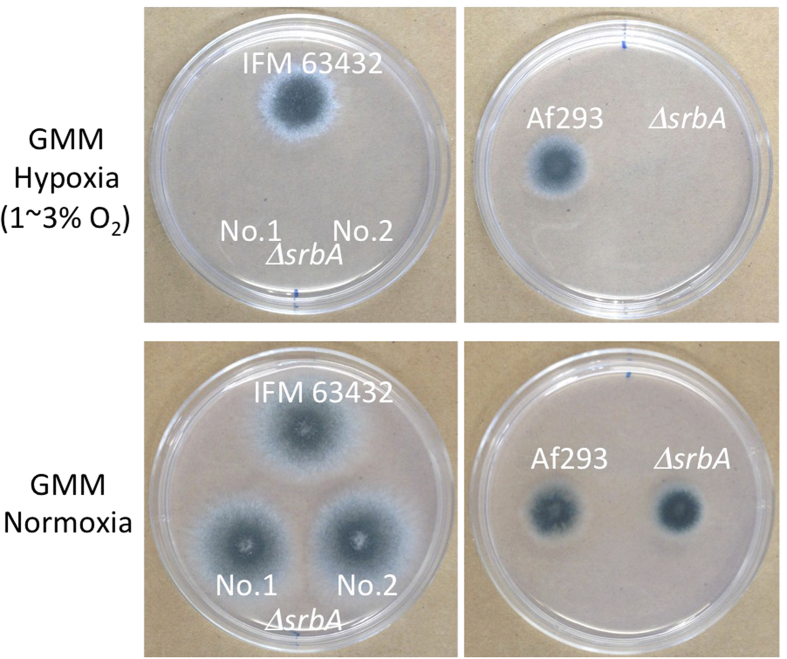
Growth under hypoxic conditions. Conidia from each strain were inoculated onto GMM, and the plates were incubated at 37 °C for 3 days in an anaeropack system whereby the concentration of oxygen was controlled at 1–3% (hypoxia). The oxygen content was monitored by oxygen analyser.

SrbA is involved in growth under hypoxic conditions^6^. Therefore, we investigated the growth of IFM 63432 and Af293 and their *srbA* deletion mutants under hypoxic conditions. In both strain backgrounds, deletion of *srbA* abolished growth under hypoxic conditions (Fig. 4).

## Discussion

The Cyp51 proteins are essential for the biosynthesis of ergosterol in fungi. Thus, azoles, inhibitors of the Cyp51 proteins, are widely used for controlling fungal growth in both agricultural and clinical settings. Some azole fungicides can inhibit human pathogens such as *A. fumigatus*^23^; however, only a few azoles are approved for use in the treatment of aspergillosis. It is becoming evident that cross-resistance to medical azoles has developed from environmental exposure to azole fungicides, resulting in azole-resistant *A. fumigatus* strains with a tandem repeat sequence in the *cyp51A* promoter region^24^,^25^. We demonstrated in the present study that an environmental azole-resistant strain became markedly sensitized to azoles following deletion of the gene encoding SrbA, a SREBP that is responsible for the regulation of *cyp51A*.

The SREBPs are a highly conserved family of transcription factors containing a basic helix-loop-helix motif, and were first identified in mammals as regulators of cholesterol metabolism^26^. These proteins are involved in the regulation of fatty acid biosynthesis and development in cholesterol auxotrophs such as *Drosophila melanogaster* and *Caenorhabditis elegans*^27^,^28^. Yeasts *Saccharomyces cerevisiae* and *Candida albicans* have no apparent SREBP orthologues, whereas *Schizosaccharomyces pombe* and *Cryptococcus neoformans* contain functional SREBPs that regulate ergosterol biosynthesis and aid in adaptation to hypoxic conditions^29^,^30^,^31^. Amongst the filamentous fungi, SrbA from *A. fumigatus* is the only characterised SREBP, except for SrbA from *Paracoccidioides*, the coding sequence of which was shown to function heterologously in *A. fumigatus* cells^32^. Bioinformatic analysis suggests that important plant pathogen species *Fusarium* and *Magnaporthe* may contain SREBPs, although the physiological functions of these proteins are yet to be investigated. Our finding of an essential role for SrbA in azole resistance in *A. fumigatus* strains containing mutations in the *cyp51A* promoter region should encourage further studies of SREBPs in other filamentous fungi. Importantly, SrbA plays a crucial role in *A. fumigatus* virulence, most likely as a result of growth impairment under hypoxic conditions. Thus, it would be of interest to investigate whether the SREBPs from plant fungal pathogens play a role in infection as well as resistance to azole fungicides.

As shown in previous studies, SrbA is involved in multiple steps in the ergosterol biosynthesis pathway, regulating the expression of *cyp51A*, *erg3B* and *erg25A*^7^,^8^. Overexpression of *cyp51A* under the control of a *niiA* promoter did not fully restore voriconazole resistance and ergosterol content to wild-type levels in an *A. fumigatus srbA* mutant strain^8^. This implied that deletion of SrbA had a multimodal effect on azole resistance in *A. fumigatus* cells. Indeed, deletion of *srbA* resulted hyper-susceptibility to azoles in a strain containing the TR46/Y121F/T289A mutations (IFM 63432), although the relative expression of *cyp51A* was still higher in IFM 63432-Δ*srbA* compared with that in the Af293 wild-type strain (Fig. 2A). The IFM 63432-Δ*srbA* strain showed decreased expression of *cyp51B*, *erg3B* and *erg25A*, the combination of which might cause the sensitisation to azoles, possibly as a result of disordered sterol profiles. Regardless, deletion of *srbA* in IFM 63432 resulted in decreased MICs for azoles, meaning that the resultant mutant strain would be susceptible to conventional azole drug therapy.

Strain IFM 63432, containing the TR46/Y121F/T289A mutations, also showed high levels of resistance to fungicide azoles such as bromuconazole, difenoconazole, propiconazole and tebuconazole. This might support the hypothesis that the azole resistance was derived from environmental exposure to fungicide azoles, as these fungicides have a similar molecular structure to the medical azoles^23^. Further occurrences and dissemination of such resistance mechanisms in the human pathogenic fungus *A. fumigatus* would undoubtedly have significant implications for public health. However, it would be virtually impossible to eliminate the use of azole fungicides in current agricultural industries. Our findings may aid in the development of alternative strategies to address the increasing threat of drug resistance. SrbA is a potential target molecule for sensitisation of resistant strains to existing azole drugs. Compounds aimed at impairing SrbA function could overcome the *cyp51A*-related azole resistance when used in combination with azoles. Additionally, the IFM 63432-Δ*srbA* mutant showed impaired growth under hypoxic conditions. As there is a close relationship between adaptation to hypoxia and fungal virulence, as shown by an attenuation of pathogenicity in a Δ*srbA* strain^6^,^31^, inhibition of SrbA might affect *in vivo* growth of *A. fumigatus*, even in the azole-resistant strains.

In conclusion, deletion of the regulator of *cyp51A* transcription, SrbA, demonstrated that an environmentally-derived azole-resistant *A. fumigatus* strain could be greatly sensitized to azoles. This suggests the possibility of developing new drugs to combat the serious threat of azole-resistant fungal pathogens.

## Methods

### Strains and growth conditions

*A. fumigatus* strains Af293 and IFM 63432 were used to generate the *srbA* deletion mutants. Clinical strain IFM 63432 contains mutations TR46/Y121F/T289A within the *cyp51A* promoter region, and was isolated in Japan in 2013^13^. All strains used in the present study were cultivated in PDA and GMM containing 1% glucose at 37 °C. Strains were grown on PDA to collect conidia. Commercially available antifungal chemicals itraconazole, miconazole, bromuconazole, difenoconazole, propiconazole and tebuconazole were obtained from Wako Pure Chemical Industries (Osaka, Japan). Iprodione, fludioxonil and kresoxim-methyl were generously provided by Dr. Keietsu Abe, Tohoku University, Sendai, Japan.

### Construction of the *srbA* deletion mutant strains

A gene replacement cassette was generated to construct the deletion mutants. DNA manipulation was performed according to standard laboratory procedures. Genomic DNA was extracted using a DNeasy Plant Mini Kit (Qiagen, Hilden, Germany). To generate a replacement cassette for *srbA*, the 5′ and 3′ flanking regions were amplified using the primers srbA-U-F(pUC119E) and srbA-U-R(ptrA), and srbA-D-F(ptrA) and srbA-D-R(pUC119B), respectively. PrimeSTAR HS DNA polymerase (Takara BIO, Otsu, Japan) was used to amplify the flanking regions from genomic DNA. Primer sequences are listed in Supplementary Table S1. These flanking regions, along with a *ptrA* fragment amplified from pPTRI (Takara BIO) using primers ptrA-F and ptrA-R, were ligated into pUC119 using a Gene Art Seamless Cloning and Assembly Kit (Invitrogen, Carlsbad, CA, USA) according to the manufacturer’s instructions. The resulting plasmid was transformed into *A. fumigatus* using a protoplast-polyethylene glycol transformation method for *Aspergillus*^33^. Precise recombination and integration were confirmed by PCR using primers Check-srbA-F and Check-srbA-R, while the absence of *srbA* transcripts was confirmed by qRT-PCR analysis (described below).

### Comparison of conidia numbers

Conidia production was examined as described previously^34^. Briefly, conidia were mixed into 2 ml of cooled liquid PDA, which was then poured into a 3.5-cm-diameter Petri plate and allowed to solidify (final concentration, 10^4^ conidia/ml). Following incubation for 72 h, the agar was transferred to a 50-ml tube containing 10 ml of PBS supplemented with 0.1% Tween 20 and vigorously vortexed to dislodge the mycelia and the produced conidia. The number of conidia in the suspensions was counted using a haemocytometer, and the number per well was calculated.

### Preparation of RNA and cDNA

Conidia from each strain were inoculated into potato dextrose broth (PDB) (final concentration, 10^5^ conidia/ml) and incubated for 16 h at 37 °C. The mycelia were frozen in liquid nitrogen, and total RNA was isolated using a FastRNA Pro Red Kit (MP Biomedicals, Santa Ana, CA, USA) according to the manufacturer’s instructions. To obtain cDNA from the total RNA, reverse transcription was performed using ReverTra Ace qPCR RT Master Mix with gDNA remover (Toyobo, Osaka, Japan) according to the manufacturer’s instructions.

### Quantitative RT-PCR

qRT-PCR was performed using SYBR Green detection as described previously^33^. Thunderbird SYBR qPCR Mix was used for reaction mixture preparation (Toyobo). Primer sets used are listed in Supplementary Table S1. The relative expression ratios were calculated using the comparative cycle threshold (Ct) (ΔΔCt) method^33^. The actin gene was used as a normalisation reference (internal control). Each sample was analysed in triplicate, and each experiment was reproduced at least twice.

### Antifungal drug susceptibility tests

#### MIC analysis

The MIC of each antifungal drug against the various *A. fumigatus* strains was investigated as described previously^35^. Tests were performed in triplicate using micafungin, amphotericin B, 5-FC, fluconazole, itraconazole, voriconazole, miconazole and posaconazole in RPMI 1640 medium (pH 7.0) at 35 °C. Assays were performed according to the Clinical and Laboratory Standards Institute broth microdilution method, document M38-A2, with some modifications (dried plates for antifungal susceptibility testing; Eiken Chemicals, Tokyo, Japan).

#### Paper disc diffusion assay

For each assay, a paper disc was placed in the centre of a PDA plate, and the test strains were inoculated by streaking conidia towards the paper disc. A 10-μl aliquot of drug solution was then dropped onto the paper disc (miconazole: 10 mg/ml; itraconazole: 10 mg/ml; bromuconazole: 10 mg/ml; difenoconazole: 10 mg/ml; propiconazole: 10 mg/ml; tebuconazole: 10 mg/ml), and the plates were incubated at 37 °C for 24 h prior to being photographed. To compare drug susceptibility by diameter of inhibition halo, conidia of each strain were mixed with 20 ml of cooled liquid GMM agar, which was then allowed to solidify in 9-cm-diameter plates (final concentration, 10^4^ conidia/ml). A 10-μl aliquot of drug solution was then dropped onto the paper disc (bromuconazole: 10 mg/ml; difenoconazole: 10 mg/ml; propiconazole: 10 mg/ml; tebuconazole: 10 mg/ml), and the plates were incubated at 37 °C for 48 h, followed by measurement of the diameter of the inhibition halo. Three replicates were carried out for each strain, and the mean and standard deviation were calculated.

### Growth under hypoxic growth conditions

Conidia of each strain were inoculated onto GMM agar plates and incubated for 72 h at 37 °C in an anaeropack system (Mitsubishi Gas Chemical, Tokyo, Japan). In this system, the concentration of oxygen was controlled within the range of approximately 1–3% (hypoxia). Control plates were incubated at 37 °C under standard oxygen conditions (21% O_2_) for 72 h.

## Additional Information

**How to cite this article**: Hagiwara, D. *et al*. Sensitisation of an Azole-Resistant *Aspergillus fumigatus* Strain containing the Cyp51A-Related Mutation by Deleting the SrbA Gene. *Sci. Rep.* **6**, 38833; doi: 10.1038/srep38833 (2016).

**Publisher's note:** Springer Nature remains neutral with regard to jurisdictional claims in published maps and institutional affiliations.

## Supplementary Material

Supplementary Table S1 and Fig S1

## Figures and Tables

**Table 1:** Antifungal susceptibility test.

Strain	MEC (mg/l)		MIC (mg/l)						
	MCFG	CPFG	AMPH-B	5-FC	FCZ	ITCZ	VCZ	MCZ	PSCZ
IFM 63432	<=0.015	0.125	1	64	>64	2	>8	2	4
IFM 63432-Δ*srbA* No. 1	<=0.015	0.25	2	>64	8	0.25	0.125	0.125	0.125
IFM 63432-Δs*rbA* No. 2	<=0.015	0.25	1	64	8	0.125	0.125	0.125	0.5
Af293	<=0.015	0.25	1	>64	>64	1	2	4	1
Af293-Δ*srbA*	<=0.015	0.25	2	64	16	0.25	0.125	0.0625	0.25

MEC: minimum effective concentration; MIC: minimum inhibitory concentration; MCFG: micafungin; CPFG: caspofungin; AMPH-B: amphotericin B; FCZ: fluconazole; VCZ: voriconazole; MCZ: miconazole; PSCZ: posaconazole.

**Table 2:** Diameters of the inhibition halos produced by fungicide azoles.

Strain	Mean diameter of the inhibition halo [mm] ± SD			
	Bromuconazole	Difenoconazole	Propiconazole	Tebuconazole
IFM 63432	0.0 ± 0.0	0.0 ± 0.0	0.0 ± 0.0	10.0 ± 0.8
IFM 63432-Δ*srbA*	>80	46.7 ± 0.5	60.7 ± 0.5	75.7 ± 1.2
Af293	33.3 ± 1.2	24.3 ± 0.5	25.3 ± 0.5	38.7 ± 0.9
Af293-Δ*srbA*	>80	78.3 ± 1.2	>80	>80
