# Supplementary material for: Sensitisation of an Azole-Resistant Aspergillus fumigatus Strain containing the Cyp51A-Related Mutation by Deleting the SrbA Gene
Source: Sci Rep. 2016 Dec 9;6:38833. doi: 10.1038/srep38833 (PMC5146965; doi:10.1038/srep38833)
Supplement: Supplementary Table S1 and Fig S1 [file srep38833-s1.pdf]

Table S1,

Sensitisation of an Azole-Resistant *Aspergillus fumigatus* Strain containing the Cyp51A-Related Mutation by Deleting the SrbA Gene

D. Hagiwara, A. Watanabe, K. Kamei

Table S1 Primers used in this study

Primer name	Sequence (5' to 3')
srbA-U-F(pUC119E)	GTAAAACGACGGCCAGTGTTCCAATGGTGTCAGATACAGATAC
srbA-U-R(ptrA)	GCCCGTCTGTCAGATCCCCATCAGATGTACTGAGAATAGTCTTG
srbA-D-F(ptrA)	CGGCTCATCGTCACCCCATGATAGTGATGATGACGCTACTGCCAGAAGC
srbA-D-R(pUC119B)	CAGGTCGACTCTAGAGATCCGAATGCCACGTCAGGGAAGAGC
ptrA-F	GGGGATCTGACAGACGGGCAATTG
ptrA-R	CTATCATGGGGTGACGATGAGCCG
Check-srbA-F	CCTTCACTTTGCGCGTTATCTCCAGG
Check-srbA-R	GCGCGTGGATACCACCGTAGTCTCG
RT-srbA-F	GTCCACCCCCGGCATTGGTGGG
RT-srbA-R	GGCAACGTCCGTACTTGATTGG
RT-cyp51A-F	GGTGCCGATGCTATGGCTTACGGC
RT-cyp51A-R	GGTTCTGTTCGGTTCCAAAGCCG
RT-cyp51B-F	GGGTCTCATCGCGTTCATTCTCGACG
RT-cyp51B-R	GATACAGCGAGGATGGATAGTAGTCC
RT-srbA-F	GTCCACCCCCGGCATTGGTGGG
RT-srbA-R	GGCAACGTCCGTACTTGATTGG
RT-erg3-F	GGATATTGTTCTTGAGATCTGGG
RT-erg3-R	CTGGTAATGAGACCGTCGAGGAG
RT-erg25A-F	GGACTCGCTCAATTCGTCCTATCCGC
RT-erg25A-R	GAGAGATGAGGTTGCTGCTGAGC
RT-actin-F	CTGTGCACATTGTCGCCAGGG
RT-actin-R	GTCCAGATTAAGCTGTCGCGC

Figure S1,
Sensitisation of an Azole-Resistant *Aspergillus fumigatus* Strain containing the Cyp51A-Related Mutation by Deleting the SrbA Gene
D. Hagiwara, A. Watanabe, K. Kamei

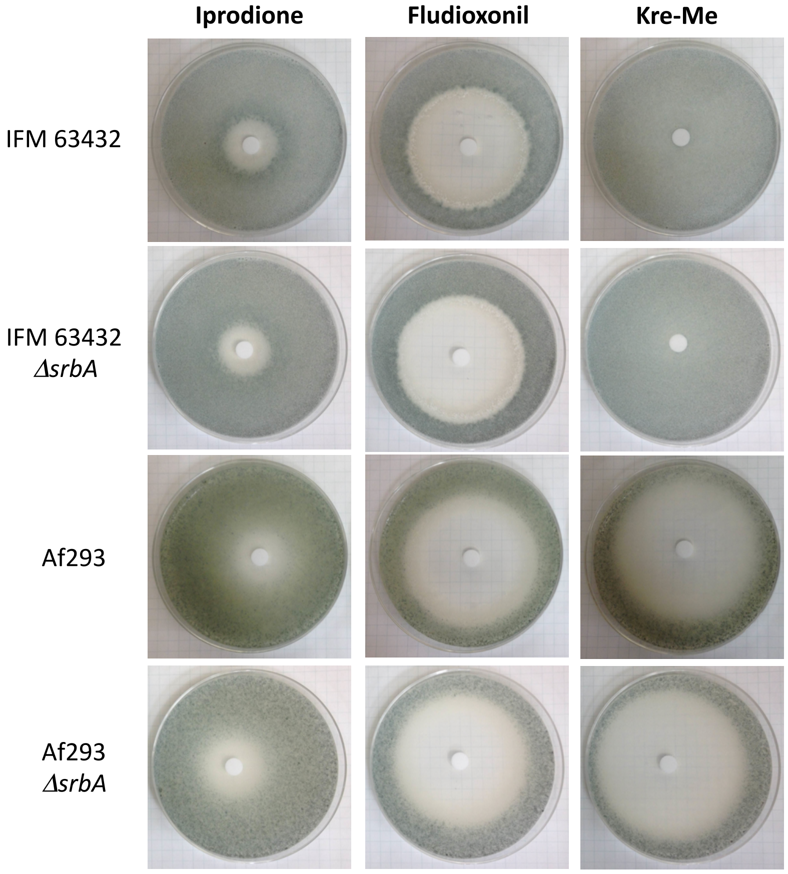

Supplemental information
Figure S1. Paper disc diffusion assays for non-azole fungicides. Conidia of each strain were mixed with 20 ml of cooled liquid GMM agar, which was then allowed to solidify in 9-cm-diameter plates (final concentration, 10^4 conidia/ml). A paper disc was placed onto the centre of each plate. A 5-ml aliquot of non-azole fungicides iprodione, fludioxonil or kresoxim-methyl (10 mg/ml) was then spotted onto the paper disc. The plates were incubated at 37°C for 48 h before being photographed.
